# Neuroinflammation in neurodegeneration *via* microbial infections

**DOI:** 10.3389/fimmu.2022.907804

**Published:** 2022-07-28

**Authors:** Van Thi Ai Tran, Luke P. Lee, Hansang Cho

**Affiliations:** ^1^ Department of Biophysics, Institute of Quantum Biophysics, Sungkyunkwan University, Suwon, South Korea; ^2^ Department of Medicine, Harvard Medical School, Brigham and Women’s Hospital, Harvard Institute of Medicine, Harvard University, Boston, MA, United States; ^3^ Department of Intelligent Precision Healthcare Convergence, Sungkyunkwan University, Suwon, South Korea

**Keywords:** infection, pathogen, multi-organ, neuroinflammation, neurodegeneration

## Abstract

Recent epidemiological studies show a noticeable correlation between chronic microbial infections and neurological disorders. However, the underlying mechanisms are still not clear due to the biological complexity of multicellular and multiorgan interactions upon microbial infections. In this review, we show the infection leading to neurodegeneration mediated by multiorgan interconnections and neuroinflammation. Firstly, we highlight three inter-organ communications as possible routes from infection sites to the brain: nose-brain axis, lung-brain axis, and gut-brain axis. Next, we described the biological crosstalk between microglia and astrocytes upon pathogenic infection. Finally, our study indicates how neuroinflammation is a critical player in pathogen-mediated neurodegeneration. Taken together, we envision that antibiotics targeting neuro-pathogens could be a potential therapeutic strategy for neurodegeneration.

## Graphical Abstract

This figure was illustrated at Biorender.com.
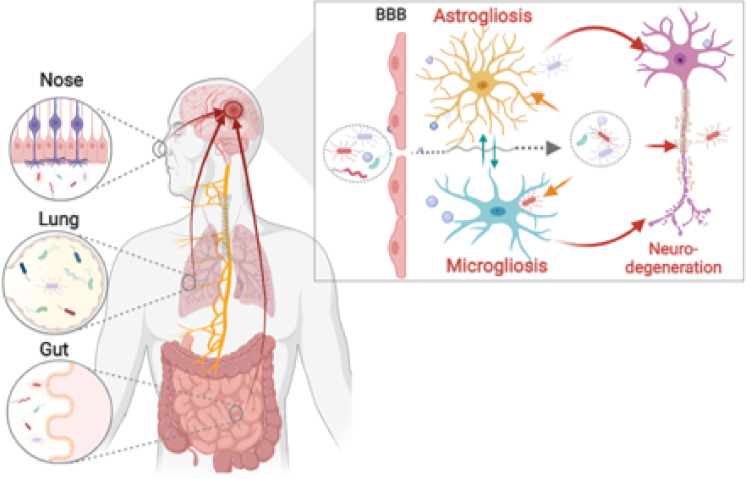

## 1 Introduction

Alzheimer’s disease (AD), Parkinson’s disease (PD), and multiple sclerosis (MS) are the most common NDs, and these affect millions of people worldwide (1). Many potential pathogenic processes of AD and PD have been explored since their discovery. The most widely accepted hypothesis relates to the aggregation of misfolded proteins such as amyloid-beta (Aβ) and tau in AD (2) and α-synuclein (α-syn) in PD pathology (3) while MS is considered an autoimmune disease (4). Currently, a new hypothesis has been proposed to indicate microbial infection as a risk factor for NDs, due to the discovery of infectious agents in the brain tissues of patients with NDs. Besides, neuroinflammation has demonstrated its potential as a central mechanism causing neurodegeneration in NDs under microbial infections (5).

For instance, decades of research have confirmed the epidemiological prevalence of bacterial infections in NDs (6–9). Microbiome-derived lipopolysaccharide (LPS) can activate microglia, leading to increased AD propagation (10, 11). Viruses are another risk factor for many neurological disorders; herpes simplex virus 1 (HSV-1) has been found in both AD and PD patients (12, 13). In addition, different brain sections of people with AD have detected the presence of fungal infections (14). Many studies have shown the involvement of microbial infections in neurological diseases; however, the role of infectious agents, including bacteria, viruses, and fungi, in NDs is still under investigation. More interestingly, the brain does not have its own microbiota, so how do the microbes reach the CNS?

Multi-organ interactions might be an answer to this question because many studies have revealed that infectious agents may be a risk factor for neuroinflammation in the CNS (15–17). The nose-brain axis is possibly the shortest pathway that allows nasal microbes to reach the brain *via* olfactory sensory neurons (OSNs), which directly contact pathogens (18), whereas the lung-brain axis is a line of connection between pulmonary microbes and NDs (19). Importantly, the concept of targeting the gut and its microbiota to heal brain diseases was presumed to be outlandish throughout the early years of the past decade (20). Since then, however, studies on brain diseases have shifted focus towards exploring the microbiota-gut-brain axis (21–23). Once microbes and their products reach the CNS, neuroinflammation is a consequence of the central immunity fighting microbial neuroinvasion, which might lead to neurodegeneration and neuron death.

Neuroinflammation is generally our defensive response against microbial infections, traumatic brain injury, or toxic aggregates while clearing wastes producing cytokines (24). Microglia and astrocytes, the most abundant brain immune cells, mainly contribute to the neuroinflammatory processes in NDs. Both alter their morphology and promote the generation of inflammatory mediators under microbial infections, which might be defined as infective neuroinflammation. Typically, their neuroinflammation has protective roles; however, certain pathogens trigger detrimental proinflammation, which could result in synaptic damage, cell loss, and entanglement of neurogenesis (25).

Therefore, this review provides a concept of infective neuroinflammation-driven neurodegeneration *via* multiorgan interconnections in NDs. We summarize the evidence supporting the infection hypothesis of NDs and discuss the latest discoveries in this field by firstly demonstrating three possible routes for the penetration of infectious agents into the CNS, including the nose-brain axis, lung-brain axis, and gut-brain axis. We then discuss the biological roles of microglia and astrocytes in the neurodegenerative neuroinflammation under microbial infections. Finally, we introduce how infective neuroinflammation-driven neurodegeneration, with a classification of the microbial infections by bacteria, viruses, and even fungi. Taken together, our review may help identify critical questions for future studies focused on an understanding of the physiology and etiology of brain disorders derived from microbes, in addition to offering a new therapeutic strategy for NDs.

## 2 Routes for microbiota invasion into the brain

### 2.1 Nose-brain axis: Olfactory pathway

**Figure 1:**
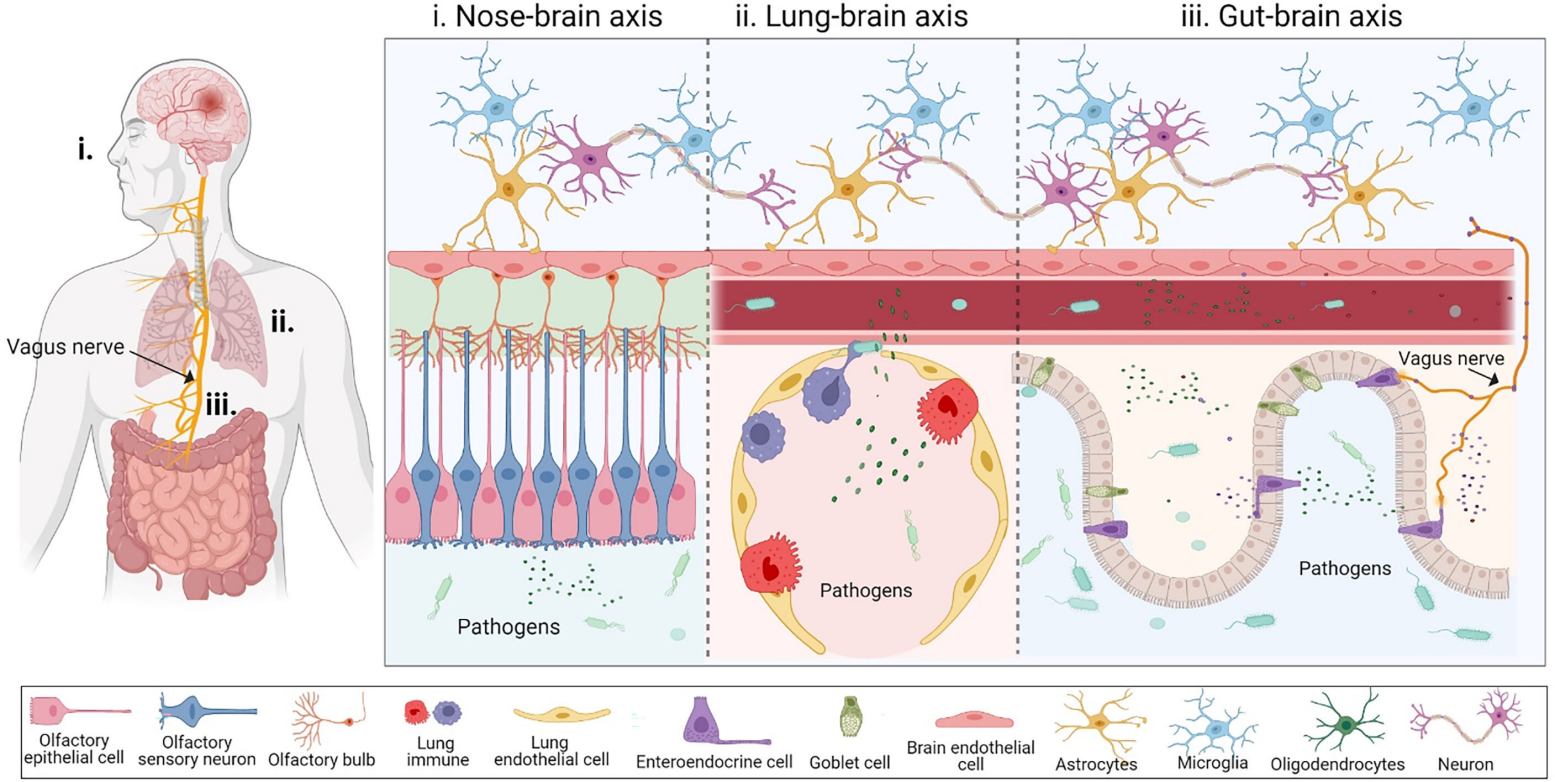
The inter-organ communication for pathogens to enter the brain. **(i)** Nose-brain axis is the first possible pathway that allows infectious agents to invade the CNS. Nasal pathogens can reach the brain by bypassing the blood-brain barrier (BBB) as well as the cerebrospinal fluid barrier. Infectious agents can invade the CNS *via* olfactory sensory neurons lying within the mucosal layer. **(ii)** Lung-brain axis: Pulmonary microbes and their soluble components may directly disrupt the lung alveolar-capillary barrier to enter the blood or interact with the local immune system, subsequently reaching the CNS by disrupting the BBB. **(iii)** Gut-brain axis serves as another compromising physiological distance connecting the gut and the brain through blood circulation and the vagus nerve. Bacteria and their components can cross the gut-blood barrier during disruption, through bloodstreams where bacteria release their metabolites, which cross the BBB and reach the central nervous system. The vagus nerve is composed of enter-endocrine cells (EECs) located on a gut sensory epithelial layer. The EEC has an extending part called a neuropod cell, which connects the gut lumen and the brain stem, allowing for bi-directional signaling. This figure was generated using Biorender.com.

The physiological distance from the nose to the brain is shorter than the gut-brain axis. However, the nasal cavity is a complicated system with many layers composed of different cells (26), including epithelium, neurons, and even immune cells (26), including epithelium, neurons, and even immune cells (**Figure 1i**). Nasal pathogens can reach the brain by bypassing the blood-brain barrier (BBB) as well as the cerebrospinal fluid barrier. Infectious agents can invade the CNS *via* OSNs within the mucosal layer, which is the most vulnerable route because it is exposed to the external environment (27). OSNs are the most abundant cell type on the olfactory epithelial surface, and their bodies are under a sheet of sustentacular cells that develop ciliated dendrites into the mucous layer (26). External odorant signals are delivered through OSN axons to the olfactory bulb of the brain (28). Due to this anatomical organization, OSNs are the direct intracellular route for neuroinvasion, and are especially vulnerable to neurotropic viruses (29).

Evidence has demonstrated that a cytopathic virus, vesicular stomatitis virus, can invade the olfactory epithelium and be transferred intracellularly along the axons of OSNs to the CNS, where the virus is first spotted in the olfactory bulb (30). In the CNS, microglia are the front-line defense against the neuroinvasion of the vesicular stomatitis virus (29). In addition, the presence of SARS-CoV-2 RNA and protein in anatomically distinct regions of the nasopharynx and brain has demonstrated the olfactory pathway as a port of SARS-CoV-2 neuroinvasion (31). Another study showed that methimazole-mediated injury led to increased *Burkholderia pseudomallei* in the olfactory system of animal models, resulting in CNS infection (32). Even though the nose-brain axis is a highly potential route to access the brain, there is still limited knowledge about this pathway, which warrants further investigation to provide a complete understanding of the underlying mechanism of how infectious agents move from the nose to the brain, causing detrimental effects.

### 2.2 Lung-brain axis

Several studies have recognized that cigarette smoking might be a risk factor for NDs, including MS (33), AD (34, 35), and PD (36), thereby suggesting a new pathway known as the ‘lung-brain axis’. Like other organs, the lungs also have a microbial community that are known as pulmonary microbes, which can directly impact the lung health and development of diseases, and indirectly be detrimental to other organs, including the brain (37). The human respiratory tract is divided into upper and lower tracts, which are primarily exposed to airway microorganisms, including bacteria, viruses, and fungi (38). Some of these reside in the alveolar zone of the lower respiratory tract. The lung-brain axis is a bi-directional association between acute respiratory distress syndrome and neurological dysfunction (39), *via* a complicated pathway consisting of inflammation and neuroendocrine pathways. Several studies have shown that multiple routes for lung infection might affect the CNS. The interaction of microbes between the lung and CNS might be through direct translocation *via* the blood circulation (40) or indirect stimulation of systemic inflammatory mediators released from the lung under infection (41), such as the cytokine storms seen in COVID-19 disease (**Figure 1ii**).

In case of direct translocation, pulmonary microbes and their soluble components may disrupt the lung alveolar-capillary barrier, to enter the blood, and then reach the CNS by disrupting the BBB. Although direct translocation is a promising route for the lung-brain axis, it has not yet been well addressed. *Pseudomonas aeruginosa*, a significant pathogen that causes pneumonia, can spread from the lungs to the bloodstream by introducing toxins to the surrounding lung epithelial cells and damaging the alveoli (42, 43). Interestingly, *P. aeruginosa* infection can cause meningitis and brain swelling (44). Another piece of evidence illustrates the regulatory roles of pulmonary microbiomes in the autoimmunity of the CNS and the development of MS (37). According to that study, the cell wall components of lung bacteria continuously send their signals to the brain immune cells and shift the polarization of the brain-resident microglial cells to a type I interferon (IFN) signature.

The direct mechanism of viral neuroinvasion is thought to be *via* peripheral sensory neurons that regulate the lung and brain by means of a neuropeptide called acetylcholine (45). Neurotropic human respiratory viruses, such as respiratory syncytial virus, remodel gene-related nerve structures in pigs (46). Among the viral respiratory infections, SARS-CoV-2 showed neurotropism for both the peripheral and central nervous systems (47), which is associated with neurological manifestations. It is known that the SARS-CoV-2 might infect the brain *via* the nasal route; however, alternative pathways have also been suggested for its neuroinvasion. One symptom found in COVID-19 patients is peripheral pain related to IFN-1-induced hyperexcitability of the dorsal root ganglion neurons, which suggests that the virus might centripetally enter the CNS through the vagus nerve (48). Subsequently, the viruses might propagate from neuron to neuron *via* synapse connection or penetrate the blood-cerebrospinal fluid barrier and damage brain cells (49, 50). In the lung-brain axis, systemic immunity might play an essential role in this crosstalk due to the inflammatory factors that are released as a defense mechanism during the infection stage (51). Upon viral infection, type-III IFNs secreted by lung dendritic cells can damage the lung epithelial layer (52). They may also play a role in BBB breakdown (53). Currently, there is much evidence showing an interconnection between SARS-CoV-2 infection and its neurological impact. Some studies have demonstrated direct neuroinvasion of the virus into the brain (31), while indirect neurological manifestations have also been indicated *via* hypercytokinemia or cytokine storms (54). The subsequent consequences of SARS-CoV-2-induced cytokines are that these travel along with the blood and disrupt the BBB, thereby damaging brain cells. Tumor necrosis factor-alpha (TNF-α) can be directly transported through the BBB, upon which it activates the inflammatory state of microglia and astrocytes, thereby inducing neuronal degeneration (55). Local lung microbiomes can be considered a warning signal for brain health, and knowledge about the lung-brain axis has the potential to serve as the basis for therapeutic strategies for NDs.

### 2.3 Gut-brain axis

Strong evidence suggests the existence of two main pathways that allow bi-directional interaction between the gut and the CNS: systemic circulation and the vagus nerve-mediated route (56, 57) (**Figure 1iii**). While these interactions are primarily beneficial, current studies have begun to focus on the relationship between the human gut and brain, which has illustrated a highly essential role of the gut microbiome as a driver of behavior, stress response, and even some brain diseases (58, 59).

#### 2.3.1 Bloodstream route: Gut-blood-brain barrier

The human gut microbiota is an intricate community of microorganisms that contains almost all bacterial species that inhabit and critically maintain homeostasis of the gastrointestinal (GI) tract (60). It can be considered “the best frenemy forever” because while it is essential and beneficial, it can also be detrimental to human health. Although most commensal microbes are dominant in the intestine and help to maintain human health, alterations in the diet or depression can modulate the composition of gut microbiota and result in an imbalance between beneficial and harmful microbes, which results in intestinal inflammation. The mucus layer, which includes a firmly adherent inner layer and a loosely adherent outer layer, serves as the first physical defense in the intestinal barrier, which prevents toxins or bacteria from directly contacting the epithelial cells (61). Thus, the first stage of chronic intestinal inflammation is dysfunction of the mucus layer under stressful conditions (62). As a result, the barrier is destroyed and pathogens attack intestinal villi and induce inflammation by producing toxins (63). *Escherichia coli* is the most common pathogenic species associated with the progression of chronic intestinal inflammation, because it contains endotoxins such as LPS, which act as inflammatory mediators in the human gut (64, 65).

Gut bacteria and their components can infiltrate gut-associated lymphoid tissues and the bloodstream, to interact with various immune cells and stimulate their responses (66). Dendritic cells that encounter translocated microbial antigens confer antigens to B and T cells, to induce their differentiation and maturation (67). The upregulation of inflammatory cytokine levels results from innate immune responses stimulated by bacterial factors (68). Abundant evidence has demonstrated that bacteria and their components can cause BBB dysfunction, which is associated with several diseases (69–71). In a rodent model of sepsis, BBB increased permeability and TNF levels (72). In meningitis, bacterial transcytosis across the BBB occurs by means of bacterial pili or cell wall components with the brain endothelium (73). CNS-tropic bacteria may penetrate the BBB without disruption, while others require it (74). Toll-like receptor (TLR)-expressing brain endothelial cells allow infiltration of LPS from gram-negative bacteria and that of lipoteichoic acid (LTA) from positive bacteria into the CNS (75). LPS can be a vital factor for stimulating inflammation of neuronal-glial cells and brain dysfunction in NDs, either alone or in combination with other potent neuropathological stimulants such as Aβ or cytokines (76, 77).

#### 2.3.2 Vagus nerve: The interface between the gut microbe and brain signaling

Initially, neurologists believed that PD starts in the CNS, and they mainly focused on α-syn as a factor involved in the pathology of PD in the brain; however, it is unknown where α-syn is present. Clinical observations have revealed that almost all patients with PD experience intestinal problems; therefore, it has been suggested that the GI tract is involved in PD. A study examining the brain of a patient with PD observed damage in the vagus nerve, accompanied by damage in the substantia nigra (SN) (78), which led to the development of a hypothesis for PD that has drawn much attention from scientists worldwide. Braak’s postulate of transportation of α-syn from the gut to the brain says that α-syn is a sub-product derived from the gut, which enters the enteric nervous system (ENS), and then travels along the unmyelinated preganglionic fibers of the vagus nerve to reach the brainstem and cause inflammation in the CNS (79–81). Proteins deposited in the brain as α-syn or Aβ can pass from one organ of the body to another. However, it is still unclear how these proteins misfold. Bacterial amyloid proteins may be involved in pathological protein misfolding in NDs (82). To investigate the function of amyloid proteins produced by microbiota, a study was conducted in rats using *C. elegans* and *E. coli* to generate curli, an amyloid protein found in the bacterial extracellular matrix. An increase in neuronal α-syn accumulation was observed in both the gut and brain of laboratory mice, whereas no differences in survival, body weight, or cytokine circulation were recorded. α-syn exposed to curli-producing bacteria also showed enhancement of α-syn propagation in a *C. elegans* model, which illustrates that bacterial amyloid may function as a trigger to initiate the deposition of aggregated proteins in the brain, *via* a mechanism called cross-seeding, which results in induced neuroinflammation (83).

With respect to the presence of α-syn in the gut, there is a proposal that enter-endocrine cells (EECs) contain α-syn and contribute to the pathogenesis of PD. EECs are sensory cells that produce hormones and connect to enteric neurons, which play a vital role in transporting proteins from the gut to the brain (84). The expression of α-syn in multiple EECs has been observed in both the small and large intestines of mice and humans. Two types of EECs, cholecystokinin and peptide YY-containing cells, have been characterized to have the presence of α-syn using several methods (85). Another animal study published evidence that PD could begin in the gut, thereby validating Braak’s hypothesis. This study injected exogenous pathological α-syn and observed its effect on the misfolding of endogenous synuclein and transmission through the vagus nerve (86). If α-syn accumulates in the gut, how does it connect the gut lumen and nervous system? Several other studies have shown that EECs possess many properties, such as, they serve as neurons, as well as express neurotrophin receptors and synaptic proteins. Furthermore, these cells possess neuropods *via* a neurofilament-containing axon-like process (87). The vagus nerve has been demonstrated to serve as a bridge that allows signals in the ENS to reach the CNS. Similarly, the dorsal nucleus of the vagus nerve has been observed in Lewy body displays, which contributes to the theory of α-syn propagation *via* this pathway (88, 89).

## 3 The roles of microglia and astrocytes in neurodegenerative neuroinflammation

**Figure 2:**
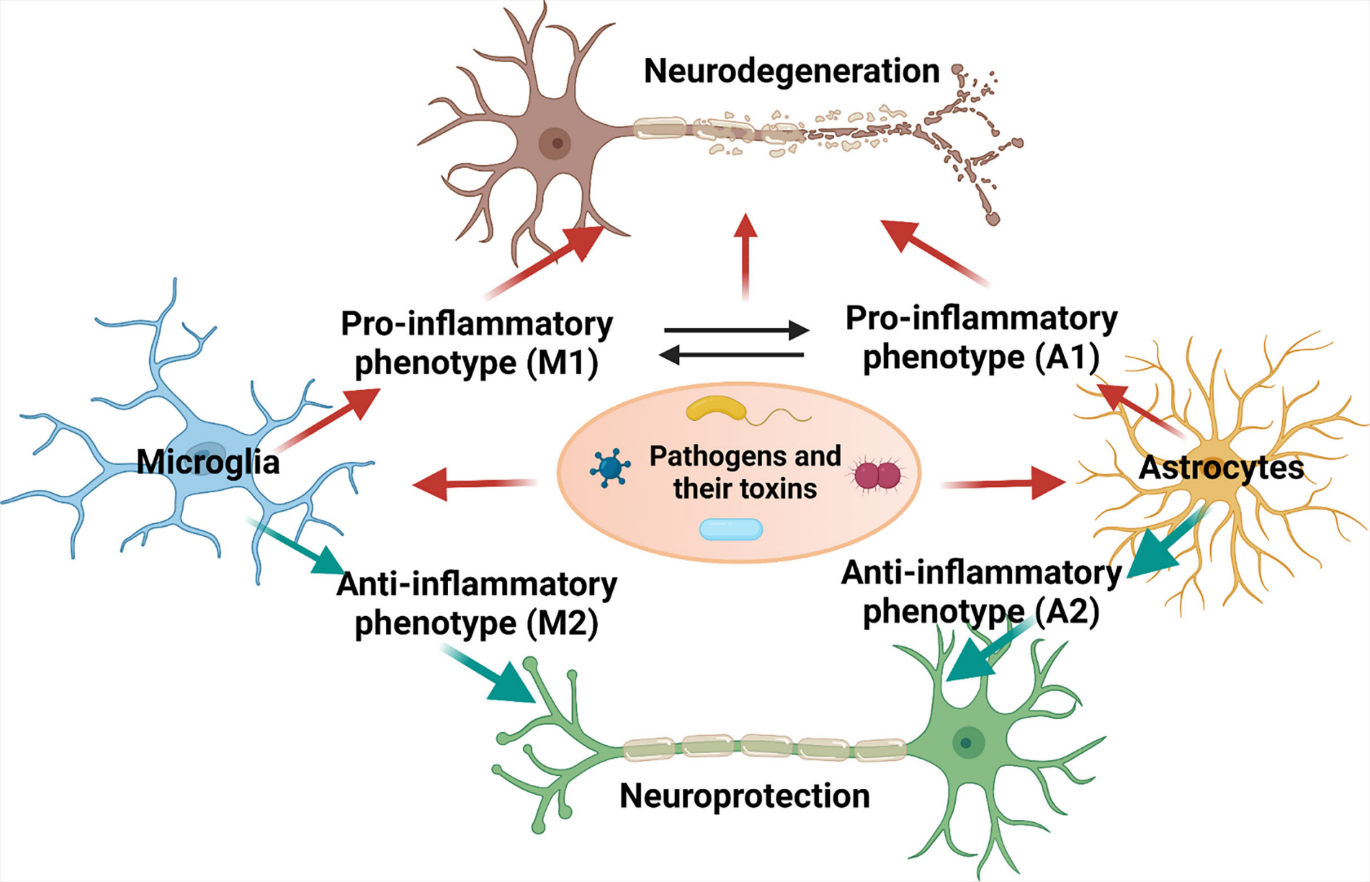
Polarization of microglia and astrocytes and their role in causing neurodegeneration upon microbial infection. This figure was illustrated using Biorender.com.

Neuroinflammation is generally defined as an inflammatory response of neuronal immune cells under various detrimental mediators such as infection, traumatic brain injury, or toxic molecules, and is indicated by the generation of several inflammatory cytokines/chemokines, nitric oxide, and ROS, by innate immune cells in the CNS (24). Microglia and astrocytes are the most abundant brain immune cells that mainly contribute to neuroinflammatory processes in NDs. Both alter their morphology, and promote the generation of inflammatory cytokines under disease conditions or infection (**Figure 2**). The production of these cytokines s could result in synaptic damage, cell loss, and entanglement of neurogenesis (25). Two prevalent cytokines found in AD, interleukin (IL)-1β and TNF-α, induce post-synaptic receptor activation and activate the nuclear factor (NF-kB) pathway, resulting in synaptic loss and neuronal death (90). Furthermore, several inflammatory cytokines in the cerebrospinal fluid are elevated in patients with NDs (91). In particular, the expression of TGF-β, MCP-1, and YKL-40 in the cerebrospinal fluid is induced in AD patients, in addition to TGF-β1, IL-6, and IL-1β in PD patients. Moreover, significant induction of G-CSF, IL-2, IL-15, IL-17, MCP-1, MIP-1α, TNF-α, and VEGF levels has been observed in amyotrophic lateral sclerosis (ALS) patients. On the other hand, the release of various anti-inflammatory cytokines, such as IL-4/10, could play a role in easing excessive chronic neuroinflammation in NDs. In addition, there are elevations in peripheral inflammatory cytokines, including IL-6, TNF, and IL-1β, in PD patients, as compared to those in controls. Taken together, peripheral and CFS cytokines may serve as biomarkers for NDs (92).

### 3.1 Microglia

Microglia are macrophage-like glial cells in the CNS immune system that have multiple vital roles in normal physiological conditions and disease progression. Their primary role is neuroprotection through the clearance of injured cells, during infection with pathogenic factors (93). Microglial activity is known to co-exist with maturation of the CNS, by adopting several regulatory pathways that contribute to the progression of NDs, such as synaptic pruning, synaptic plasticity preservation, neuronal apoptosis, and immune surveillance (94). Microglia can effectively recognize and respond to harmful pathological mediators, such as pathogens or abnormal proteins, *via* pathogens/damage-associated molecular patterns, which are surface receptors of microglia. Stimulated microglia can degrade pathogenic factors *via* phagocytosis or activation of chemokine receptors and IFNs, which are the main components of the neuroinflammatory process (95). This activity cannot be maintained sustainably in microglia of aged brains, due to functional impairment, thereby increasing the risk of the pathogenesis of NDs.

Microglia have a complicated action in the orientation of NDs, because of their various phenotypes and different activation pathways. Microglia show morphological alterations in aging brains with decreased branches, leading to reduced surveillance and further promotion of homeostatic dysfunction. In addition, microglial phenotypes vary in different brain regions and with disease progression; indeed, Aβ plaque-associated microglia show significant morphological changes (96). Moreover, microglia in the late disease state show a more profound phenotypic alteration than those in the mild stage. Phosphorylated tau (p-tau) protein can promote microglial phenotypic alteration, resulting in loss of immune surveillance, and is associated with AD progression *via* the formation of neurofibrillary tangles (NFTs) (97). Based on this evidence, it is believed that changes in microglial phenotype could facilitate progression to the AD stage.

In the same case of astrocytes, activated microglia are divided into two main groups: M1 activation expresses inflammatory characteristics, while M2 activation represents anti-inflammatory activities. Transcriptomic analysis of AD mice revealed that a gradual transition of microglia from a disease-associated state is regulated by the downregulation of homeostatic genes and upregulation of Apolipoprotein E (APOE) and triggering receptor expressed on myeloid cells 2 (TREM2), which emphasizes the essential roles of TREM2 in AD pathogenesis (98). Furthermore, temporal imaging of the mouse AD brain demonstrated different gene markers for microglial activation in distinctive states. For example, the mild state is marked by proliferation-associated genes, whereas immune response genes indicate the late state of the disease (99). The exact role of the microglial phenotype and its contribution to disease progression are currently being unraveled, to determine the heterogeneity of microglia in aging brains.

### 3.2 Astrocytes

Astrocytes are the most common glial cells in the CNS, which help maintain the BBB function, and support synaptic activities by releasing neurotransmitters and eliminating neurotoxic molecules, thus helping in the maintenance of a healthy brain (100). Reactive gliosis is the response of astrocytes to pathological mediators that contribute to neuroinflammation. Reactive astrocytes express glial fibrillary acidic protein (GFAP), which is an important marker for determining the status of astrocytes in a disease condition. Inflammatory factors have been shown to activate the NF-kB signaling pathway, leading to the A1 phenotype of astrocytes, while ischemia stimulates the A2 state of astrocytes *via* signal transducer and activator of transcription 3 (STAT3) transcription factor (101). A1 astrocytes produce more inflammatory mediators that are significantly detrimental to neuronal cells, whereas the A2 phenotype serves a protective role by generating neurotrophic factors.

Given their various housekeeping activities, astrocytes are expected to restore homeostasis in the mild stage of AD. For example, astrocytes containing Aβ-positive granules were found in the human brain, indicating a contribution of astrocytes in clearing harmful forms of Aβ during disease progression (102). In addition, astrocytes are recruited to the brain region containing Aβ plaques, to degrade abnormal Aβ deposition. Despite the various beneficial roles of astrocytes, the A1 phenotype found in brain tissues of AD patients showed toxicity of astrocytes towards neurons, by releasing a massive amount of gamma-aminobutyric acid (GABA) and glutamate, resulting in synaptic dysfunction and memory impairment (101). Furthermore, A1 astrocytes also showed their ability to disrupt the BBB associated with amyloid protein aggregation, which suggests the pathological role of A1 cells in the generation of Aβ during the early progression of AD (103). Another critical point is that astrocytes could mediate the detrimental activities of microglia in AD states, due to their related roles.

## 4 Infective neuroinflammation, and neurodegeneration

### 4.1 Bacteria-driven neurodegenerative neuroinflammation

#### 4.1.1 Intestinal bacteria

Targeting the gut and its microbiome to treat brain diseases may have been considered outlandish throughout the early years of the past decade (20, 104). However, it has rapidly gained much attention since 2004, when a correlation between gut bacteria and stress response in a mouse model was reported, which indicated that common microbes in the GI tract could play a role in the post-natal development of the hypothalamic-pituitary-adrenal stress response (105). The gut microbiota is the most critical and complex microbial habitat in the human body, with an estimated ratio of around 10:1 microbial cells to human cells (60, 106, 107). Furthermore, an abundance of publications have revealed that the intestinal microbiome significantly affects the pathogenesis of multiple neurological diseases (**Table 1**) (108).

Interestingly, alterations in the components of intestinal bacterial species have been found in APPPS1 mice, with a reduction in several phyla, including *Firmicutes*, *Actinobacteria*, *Verrucomicrobia*, and *Proteobacteria*. In contrast, members of *Bacteroidetes* and *Tenericutes* were found to be significantly elevated. More importantly, APPPS1 transgenic mice raised in germ-free conditions partly inhibited the generation of aggregated Aβ, due to increased production of enzymes degrading Aβ, which suggests that the complete knockout of microbiota does not completely inhibit the development of Aβ (109). The involvement of bacteria in the progression of AD has been demonstrated in several studies. The use of antibiotics leads to a reduction in Aβ toxicity and plaque formation, while enhancing memory and learning abilities. Rifampicin and minocycline, which can penetrate the BBB, are the two most popular antibiotics used in studies associated with AD, in both human and mouse models. The administration of rifampicin and minocycline reduces Aβ levels and toxicity, tau phosphorylation, neuroinflammation, in addition to enhancing memory in APP_OSK_ mice (110–112). Furthermore, long-term treatment with an antibiotic cocktail reduced circulating inflammatory cytokines and chemokines in APP_SWE_/PS1_△E9_ double transgenic mice (113). *Helicobacter pylori*, an important pathogen of the GI tract, is the leading cause of inflammation in the gastric lumen. Infection of *H. pylori* is a high-risk cause of AD. A study using rat models showed memory loss and spatial learning defects in hippocampal dendritic spine cells, which might be due to the induction of Aβ_42_ levels, by enhancement of γ-secretase during *H. pylori* infection (114).

In addition, human studies have demonstrated a co-operative relationship between gut bacterial dysbiosis and AD. An established correlation between irritable bowel syndrome and a high risk of dementia suggests that gut bacterial imbalance can be mediated by this association (115). However, the abundance of phyla in the bacterial community is still debated, owing to several inconsistent studies, which could be attributed to the diversity in regions, diet, and the original structure of the bacterial community. Stool samples from patients with cognitive impairment and amyloidosis have been shown to display an increased proportion of harmful bacteria, such as *Escherichia* and *Shigella*, in addition to a reduction in the proportion of beneficial bacteria. These changes were associated with the induction of circulating inflammatory markers, leading to the hypothesis that gut dysbiosis may result in systemic inflammation and further contribute to disease progression. Various *in vitro* studies have demonstrated the detrimental role of LPS, an element of the bacterial outer membrane, in amyloid protein accumulation, and that bacterial extracellular DNA can induce NFT formation (116, 117). The deleterious role of LPS, which enhances neuroinflammatory responses and accumulation of neuronal amyloid protein, has been further confirmed in the APPwe transgenic mouse model (118). Intriguingly, LPS levels in human AD brains are elevated and co-localized with Aβs. Probiotics have been utilized in AD treatment, and an enhancement in mini-mental state examination scores has been recorded after 12 weeks of probiotic supplementation (119).

**Table 1:** Association of bacterial infection and neurological disorders.

Names	Human organs	Associated features	References
*Helicobacter pylori*	Gastric lumen	APP, APOE, PSEN1, and PSEN2	(120, 121)
*Escherichia coli*	Intestine	BBB invasion Neuroinflammation Neurodegeneration	(122–124)
*Porphyromonas gingivalis*	Oral cavity	Aβ and p-tau Neurodegeneration Neuroinflammation	(17, 125, 126)
*Chlamydia pneumoniae*	Nasal cavity	BBB alteration Neuroimmune responses	(127, 128)
*Mycobacterium tuberculosis*	Lung	BBB disruption Neuroinflammation	(129, 130)
Lipopolysaccharides		BBB disruption Neuroinflammation Aβ and p-tau	(131–133)
Lipoteichoic acid		BBB disruption Neuroinflammation	(134–136)
Short-chain fatty acids		Amyloid pathology Autoimmune neuroinflammation	(137, 138)
Bacterial DNA		Aβ and Tau aggregation	(117, 139)

#### 4.1.2 Oral bacteria

The oral cavity harbors over 700 bacterial taxa, primarily *Bacillus*, *Firmicutes*, *Actinomycetes*, and *Proteobacteria* (140). Most oral bacteria are anaerobes residing on the subgingival surface, known as the dental plaque; therefore, daily removal of the plaque by brushing teeth is essential to prevent disease (141). However, some bacteria can escape from the oral cavity and potentially have distinctive colonization in other organs, such as the heart; for example, infective endocarditis might also be invasive in the brain (**Table 1**). The development of chronic periodontitis, commonly known as gum disease, is a crucial consequence of a microbial imbalance in the oral cavity. Periodontal diseases destroy periodontal tissues and are attributed to several systemic diseases. Gum disease has recently been shown to be correlated with the progression of AD, and periodontitis patients have shown the induction of Aβ deposition in the brain (142, 143). A study found that *Carbachia*, *Clostridium*, *Porphyromonas*, *Helicobacter*, *Actinomycetes*, *Eugenia*, *Tannella*, *Hurdella*, *Micromonas*, and *Streptococcus pneumoniae* were much more abundant in the oral microbiota of periodontitis patients than in healthy individuals. Among the bacterial genera associated with gum disease, *Porphyromonas gingivalis* has been highlighted for its detrimental role in causing features of AD (17).

*Porphyromonas gingivalis*, a member of the Bacteroidetes phylum, is a gram-negative bacterium that can cause inflammation and generate toxins that destroy the tissues supporting the tooth (144). Cysteine protease or gingipains, the main toxins produced by *P. gingivalis*, can escape immune detection by suppressing adaptive immunity. Furthermore, it has been proposed that the association of immune suppression and gingipain-induced tissue destruction enables bacteria to leave the oral cavity and migrate to the CNS, where they can reside and cause AD through gingipain activity. Various studies have shown the co-localization of *P. gingivalis* and the Aβs, both *in vivo* and in the human brain (145–147). A recent investigation illustrated that oral injection with *P. gingivalis* in mice could result in bacterial colonization of the brain, induction of Aβ aggregation, and p-tau protein (126). Gingipain inhibition reduced several hallmarks of AD in a mouse model, such as the formation of Aβ plaques and tau tangles as well as neuroinflammatory responses and neuronal loss. The recent discovery of small-molecule inhibitors of gingipains has introduced a novel approach to advance our understanding of bacterial infection and AD. It has been found that there is an induction of serum antibodies marked for several periodontal disease-associated bacteria, such as *Prevotella intermedia*, *Actinomyces naeslundii*, and *Eubacterium nodatum*, in AD samples, even before the onset of AD (142, 148). In addition, spirochetes, another main cause of periodontitis, have been shown to be associated with AD, because of their ability to penetrate the CNS (149). The immunological and genetic materials of oral Treponema species, *Treponema pectinovorum*, and *Treponema socranskii*, were also found in the brain specimens of AD patients (150). Based on the significant correlation between several oral bacteria and AD, oral bacteria may play a detrimental role in disease progression.

#### 4.1.3 Nasal and lung bacteria

Similar to other regions of the human body, the upper respiratory tract comprises diverse bacterial species, including beneficial and pathogenic strains that contribute to human health (**Table 1**). Many studies have indicated the predominance of *Bifidobacterium*, *Staphylococcus*, and *Streptococcus* in healthy humans (151, 152). Some reports have demonstrated the potential of nasal bacteria to cause NDs, such as *Chlamydia pneumoniae* causing AD (153) and *Staphylococcus aureus* causing MS (154). In addition, olfactory dysfunction can be an early symptom of PD and the initial stage of α-syn pathology (78). However, the involvement of nasal bacteria and NDs has not yet been explored in detail in the case of AD or MS, although a few studies have revealed the prevalence of nasal bacteria and olfactory deficits in PD. While there were no significant differences between the components of nasal bacteria in PD and healthy people, a study reported that two taxa of bacteria, *Flavobacteriaceae* and *Marmoricola*, were less abundant in the PD group than in the control group (155). Another study suggested that nasopharyngeal bacteria may incite rebellion of innate immune system priming, which may induce the development of misfolded proteins and oxidative stress in the CNS (156).

*Mycobacterium tuberculosis*, a well-known cause of tuberculosis, is a major invader of the CNS. *M. tuberculosis* infection can activate the early response of TLR signaling, to form myddosomes, and in addition, rearrange the cytoskeleton in brain endothelial cells, leading to BBB disruption (157, 158). TNFs, the main inflammatory cytokines, are released by the innate immune system during *M. tuberculosis* infection. However, TNFs are also critical mediators that enhance the generation of amyloid proteins and their accumulation, subsequently reducing phagocytic function and further increasing the loss of neuronal cells, which are essential features in the pathogenesis of AD (159). During infection, there is an increase in neuroinflammation by the altered presence of endothelin-1 (ET-1), which is mainly produced by endothelial cells to maintain the function of the BBB. Although ET-1 usually acts as a vasoconstrictor, it is also an inflammatory cytokine that can induce the aggregation of platelets and production of leukocyte adhesion molecules. In addition, the generated cytokines can stimulate vascular dysfunction and inflammatory responses in the CNS (160). ET-1 overexpression has been associated with various infectious diseases, suggesting a relationship between infectious diseases and neuroinflammation. The induced level of ET-1 has also been shown in neurological diseases, such as PD (161) and AD (162). Taken together, the hypothesis that infection may mainly contribute to the progression of brain diseases is becoming more evident, particularly in AD.

#### 4.1.4 Bacterial components

The association between LPS levels in the brain and the pathological development of AD has been demonstrated by observing the abundant presence of LPS in the neocortex and hippocampus of AD brains. LPS also has an adherent attachment to the nuclear periphery in AD brain cells (76). The brain and blood levels of many types of cytokines were elevated after LPS injection in male Sprague Dawley rats. In addition, there was an increase in the levels of soluble Aβ and p-tau in the whole brain within seven days, which indicates the possibility of downstream consequences of Aβ formation, and also serves as evidence that LPS reaches the CNS through blood circulation (163). Moreover, LPS can affect misfolded α-syn formation and dopaminergic neurodegeneration, the main hallmarks of PD pathophysiology. A loss of dopaminergic neurons within four days induced up to 34% loss in the SN, as compared to that in the control treatment (164). In addition, LPS can bind to α-syn, to initiate and proliferate amyloidogenesis in the gut, and then transport it *via* the vagus nerve to the CNS (165). ALS and Huntington’s disease (HD) are two other neuronal disorders in which LPS affects the pathophysiology. ALS is a disease that affects the voluntary motor system and is characterized by the degradation of spinal cord motor neurons. The gene expression of a protein associated with ALS was increased upon LPS injection, and it has been observed that the activation of astrocytes and microglia increases with LPS-induced inflammation in the ALS model (166). The other neurodegenerative disorder, HD, is characterized by the presence of motor, cognitive, and behavioral dysfunction. Few studies have investigated how inflammation affects the neurodegeneration in HD, but it has been shown that a peripheral injection of LPS stimulated microglial alterations and vascular dysfunction in a model of 12-month-old YAC128 transgenic mouse (167).

*In vitro* experiments have employed LPS, an endotoxin presents in the outer membrane of numerous gram-negative bacteria, to mimic bacterial infection. LPS can bind to TLR-4 expressed on the surface of microglia and other immune cells, to enter the cytosol, where it activates associated inflammatory responses (168). Communication between TLR-4 and LPS initiates the formation of a myddosome composed of various proteins. The myddosome structure can activate the NF-kB signaling pathway, further stimulating several inflammatory genes (168). Interestingly, NOD-1 and NOD-2, two primary nucleotide binding oligomerization domain (NOD)-like receptors, can detect elements of the bacterial cell wall and stimulate NF-kB and MAP kinase-dependent inflammatory responses (169). Furthermore, an *in vivo* study indicated that co-stimulation of NOD- and LPS-activated TLR-4 affected brain function and sickness behavior (170).

Epilepsy, which is an example of infection-mediated neuroinflammation, is characterized by spontaneous seizures in the brain. A positive association between infection, neuroinflammation, and epilepsy has been confirmed by means of imaging studies of the human brain (171). Bacterial LPS can initiate epilepsy in *in vivo* models, *via* the activation of IL-1β (15) and cyclooxygenase-2-dependent inflammation (172), which indicates a high susceptibility to seizures and a strong oxidative response during LPS-mediated neuroinflammation (173). In addition, LTA, a significant component of the cell wall of gram-positive bacteria, supports the binding of bacteria to brain microvascular endothelial cells (174). LTA has been detected in mouse brain samples and is correlated with the levels of IFN-γ, IL-6, and other cytokines. In the brain, LTA is also related to the overexpression of circulating corticosterone and reduction of tight junction proteins expressed in the BBB layer (135). Upon the onset of bacteriolysis, LTA is produced in circulation and it binds to TLR-2, to trigger the release of several inflammatory cytokines, causing BBB disruption and neuroinflammatory responses in the CNS (134).

### 4.2 Viruses-driven neurodegenerative neuroinflammation

The virus is a hazardous infectious agent that causes many pandemic outbreaks, such as COVID-19, AIDS, and Ebola. Several brain dysfunctions have been observed in patients with viral diseases, suggesting that viral infection is a risk factor for neurological disorders (**Table 2**) (175). Different viruses have been shown to correlate with the pathology of brain diseases. Enterovirus and human herpesvirus are associated with ALS (176, 177), while Epstein-Barr virus (EBV), cytomegalovirus, and varicella-zoster virus have been reported in MS patients (178, 179). In addition, Japanese encephalitis virus (JPV) and influenza virus have been identified in patients with PD (180), while three strains of human HSV have been found in the brain samples of AD patients (181–183). Several studies have indicated viral neuroinvasion and damage to neural cells in the CNS, either directly or indirectly, by stimulating neuroinflammatory responses (184).

In the case of neurotropic viruses, some influenza strains invade the brain *via* different cellular routes, either by infecting brain endothelial cells or through the nerve network (the olfactory or vagus nerves) (185), followed by polarization of microglia in the central innate immunity (186). For instance, neurotropic H7N7-infected mice displayed increased inflammatory gene markers of activated microglia and loss of neurons in the hippocampus. In particular, the major histocompatibility complex II (MHC-II) was strongly expressed, demonstrating a direct interaction between microglia and the virus. Increased MHC-II expression is a general feature of microglial activation and inflammation (187), which is also associated with MS pathogenesis (188). Furthermore, Sic6a3-associated neuropsychiatric disorders are upregulated after long-term H7N7 infection (189). Another strain of influenza virus, H1N1, also showed detrimental effects on the microglia and neurons in mice, as defined by the high levels of inflammatory cytokines (IL-1β, IL-6, TNF-α, and IFN-α) and markers for stimulated microglia, in addition to changing the morphological characteristics of the hippocampal neurons (186).

Currently, it has been confirmed in clinical studies, animal models, and cellular models that SARS-CoV-2 is involved in neuroinvasion and the associated toxicity *via* neuroinflammation and neuronal death (190–192). A single-cell transcriptomics study revealed the strongest alteration of inflammatory-related genes in astrocytes and glial cells, by means of RNA sequencing and staining of the brain tissues of people who died from COVID-19 (193). For example, increased levels of chitinase 3-like 1, GFAP, and interferon-induced transmembrane protein 3 are found in astrocytes, whereas CD14, CD74, and CTSB are induced in microglia. CHI3L1 is considered a biomarker in mild stages of MS, and a high level of CHI3L1 in the cerebrospinal fluid is associated with the development of neurological disorders (194). In addition, neuronal degeneration and apoptosis are induced by SARS-CoV-2 infection, along with neuroinflammation (192). Based on this evidence, neuroinflammation may play a central role in neuronal death during influenza virus infection.

In addition, human HSV may be another potential viral agent that contributes to the inflammatory pathways of NDs (195–198). HSV infection increases encephalitis, which is characterized by severe neuroinflammation and prolonged neurological deficits (199). Microglia have been suggested as key players that fight against HSV infection, by releasing IL-10, an anti-inflammatory cytokine, which suppresses HSV-triggered neuroinflammation in microglial cells (195). In contrast, human microglial cells respond to HSV-1 by generating inflammatory cytokines/chemokines, including TNF, IL-1β, CCL5, and CXCL10. HSV can bind to TLR2 expressed in the microglia and astrocytes, to induce inflammatory cytokines, including IL-6 and IL-1β, which are associated with an increase in detrimental misfolded proteins that serve as neurodegenerative markers (196, 197). HSV-1 microglial infection induces inflammatory cytokines and Inducible nitric oxide synthase by downregulating Fas and upregulating the FasL signaling pathway (198). Moreover, co-localization of NO production and Aβ accumulation has been found in HSV-1-infected neurons, *in vitro* and *in vivo* (198). EBV is a member of the herpes virus family; it causes mononucleosis and is found in MS, which is defined as a chronic neuroinflammatory condition within the brain (200). Intravenous peripheral EBV-infected cells break down the BBB, infiltrate the CNS, and trigger neuroinflammation in the rabbit brain (201). Cytomegalovirus, which is also a cause of chronic immune activation in MS, drives autoimmune-mediated neuroinflammation and demyelination (202, 203).

**Table 2:** Association of viral infection and neurological disorders.

Viruses	Associated features	References
**SARS-CoV-2**	Brain invasion BBB disruption Microglial activation	(204–206)
**Influenza virus**	Neuroinvasion BBB disruption Neuroinflammation	(189, 207, 208)
**Human herpes simplex virus**	BBB apoptosis Neuroinflammation Neurodegeneration	(198, 209, 210)
**Epstein-Barr virus**	Neuroinvasion BBB disruption Neuroinflammation	(200, 201, 211)
**Japanese encephalitis virus**	BBB disruption Neuroinflammation Neuron infection	(212–214)
**Zika virus**	Neuroinvasion Brain structure alteration Impairment of synapse	(215–217)

Another dangerous neurotropic RNA virus is the Japanese encephalitis virus (JPV), which is transmitted by mosquitoes. Significantly, this virus can disrupt the BBB after four days of infection, with viral titers found in the brain on day two, accompanied by a high level of cytokines, indicating inflammation-mediated BBB disruption (212). Another report pointed out that the NLRP3 inflammasome is a key player in the neuroinflammatory response to JPV infection in the microglia, which is characterized by increased levels of IL-1β and IL-18 (218). Microglia recognize the JPV *via* the TLR3 and TLR4 signaling pathways, causing neuroinflammation and leading to neurodegeneration (219). The Zika virus (ZIKV) is a neurotropic virus that induces adult neuropathy. ZIKV infection causes an increase in CXCL12, which regulates lymphocyte trafficking through the BBB (220). After infecting brain microvascular cells, ZIKV is released on the parenchyma side, and it initiates the alteration of BBB integrity and upregulation of inflammatory and cell adhesion molecules (221). In the CNS, microglia and astrocytes are involved in ZIKV replication and elimination (222).

### 4.3 Fungi-driven neurodegenerative neuroinflammation

Approximately 300 out of the 70,000 described fungal species may be detrimental to human health, and approximately 10% of these 300 species influence the brain (223). However, the pathological effects of fungi on the CNS have not been fully explored (**Table 3**). Proteomic and genomic studies have indicated the presence of fungal proteins and DNA in the brain tissues of AD patients (14). Further evidence indicated the existence of several fungal species in AD brains, by means of immunohistochemistry and PCR analysis (14, 224). In addition, a comprehensive analysis of PD samples identified that most of the fungal genera *Botrytis*, *Candida*, *Fusarium*, and *Malassezia* are accompanied by bacterial species in the CNS, which suggests that mixed infection with bacteria and fungi may be risky for PD pathology (225). The fungal genus *Malassezia* has also been detected in patients with MS (226), and may use macrophages to reach the CNS (227).

**Table 3:** Association of fungal infection and neurological disorders.

Fungi	Associated features	References
*Cryptococcus neoformans*	BBB invasion Microglial phagocytosis	(86, 228, 229)
*Candida albicans*	Brain invasion IL-1β, IL-6, and TNF production Memory deficits	(230–232)
*Aspergillus fumigatus*	Brain invasion BBB integrity impairment	(233, 234)
*Malassezia*	Brain invasion	(225)

The BBB is the most promising route for the penetration of fungi into the CNS, *via* transcellular migration, paracellular migration, and the Trojan horse mechanism. Transcellular and paracellular migrations are direct ways to pass the BBB, *via* transcytosis of endothelial cells, while Trojan horses are related to transport-mediating phagocytosis (235–237). The translocation of fungi occurs *via* transcellular and paracellular mechanisms, which requires contact between fungal proteins and the BBB. For instance, activation of protein kinase C-alpha mediates the transcellular transport of *Cryptococcus neoformans* through brain microvascular endothelial cells (229). Agglutinin-like protein precursor (ALs3), a cell surface adhesion protein, interacts with a heat shock protein of the brain endothelium to initiate the internalization of *Candida albicans* (238). Several studies have demonstrated that T cells, endothelial cells, microglia, and astrocytes play important roles in inhibiting fungal proliferation, by releasing cytokines, nitric oxide, superoxide, and MHC-I/II molecules (239). These cells may recognize fungal antigens, such as polysaccharide capsules (*C. neoformans*), pseudohyphae (*C. albicans*), or conidia (*Aspergillus* spp.), *via* TLR-2, -4, or -9, while Dectin-1 and Complement receptor 3 may sense the presence of fungal surface carbohydrates such as mannose in *Aspergillus fumigatus* and β-glucans in *C. albicans*.

Fungal infection can be controlled by initiating pro-lymphatic and humoral response-induced microglial activation (240, 241). For example, microglial cell-expressed TLR-4 is predominantly present on the surface, and its interaction with fungal antigens induces inflammatory responses that mediate T helper type 1 development to fight against fungi (242). TLR-4 knockout mice are more susceptible to fungal infection and have reduced clearance of *Aspergillus* (243). Microglia can control fungal growth by producing anti-inflammatory chemokines, such as CCL2, to increase animal susceptibility to *Cryptococcus neoforman* infection (244). The anti-inflammatory cytokine IL-10 is released at a high level, to modulate *C. albicans* infection (245).

## 5 Outlook

In this review, we summarize the possible axes for microbial invasion into the CNS and the current discoveries connecting three factors involved in NDs: microbial infection, neuroinflammation, and neurodegeneration. Pathogens can reach the brain *via* the olfactory system, blood circulation, and vagus nerve pathway. In the CNS, neural immune cells can be stimulated upon infection and induce inflammatory responses, causing neuroinflammation, which further leads to neuronal death. However, there is still a limited understanding of which pathogens play a dominant role in neuroinflammation and neurodegeneration, because of the lack of relevant human models to adopt complicated physiological features. Therefore, it is necessary to develop human cellular platforms to study the cellular mechanisms of microbial neuroinvasion, determine the risk factors for NDs, and provide promising tools for discovering new treatments.

## Author contributions

VT designed/generated the figures and wrote/edited the manuscript. HC conceived the idea, provided guidance, and edited the manuscript. LL guided and edited the manuscript. All the authors have read and agreed to the published version of the manuscript.

## Funding

This study was supported by the National Research Foundation (grant numbers: NRF-2020R1A2C2010285, NRF 2018M3C7A1056896, and NRF- I21SS7606036) to HC.

## Conflict of interest

The authors declare that the research was conducted in the absence of any commercial or financial relationships that could be construed as potential conflicts of interest.

## Publisher’s note

All claims expressed in this article are solely those of the authors and do not necessarily represent those of their affiliated organizations, or those of the publisher, the editors and the reviewers. Any product that may be evaluated in this article, or claim that may be made by its manufacturer, is not guaranteed or endorsed by the publisher.
